# High glucose inhibits receptor activator of nuclear factor-κB ligand-induced osteoclast differentiation via downregulation of v-ATPase V0 subunit d2 and dendritic cell-specific transmembrane protein

**DOI:** 10.3892/mmr.2014.2807

**Published:** 2014-10-29

**Authors:** JUAN XU, FENG YUE, JINGBO WANG, LI CHEN, WENBO QI

**Affiliations:** 1 Department of Endocrinology, Qilu Hospital, Shandong University, Jinan, Shandong 250012, P.R. China; 2 Department of Endocrinology, Taian City Central Hospital, Taian, Shandong 271000, P.R. China; 3 Department of Central Laboratory, Taian City Central Hospital, Taian, Shandong 271000, P.R. China

**Keywords:** diabetes, osteoclast differentiation, v-ATPase V0 subunit d2, dendritic cell-specific transmembrane protein

## Abstract

The balance between bone formation and resorption is compromised in diabetes, which may contribute to the high risk of fractures in diabetic patients. However, the mechanism by which high glucose affects bone turnover remains to be elucidated. The present study demonstrated that high glucose inhibited receptor activator of nuclear factor-κB ligand (RANKL)-induced osteoclastogenesis. In order to examine the mechanism involved in the inhibition of osteoclastogenesis, the present study examined several key molecules involved in osteoclast differentiation, including v-ATPase V0 subunit d2 (Atp6V0d2), dendritic cell-specific transmembrane protein (DC-STAMP), c-fos and nuclear factor of activated T cells c1 (NFATc1). The expression levels of Atp6V0d2 and DC-STAMP are regulated by NFATc1 and c-fos, and are required for osteoclast fusion, which is important for osteoclast maturation. To the best of our knowledge, the present study demonstrated for the first time that high glucose decreased the gene expression of ATP6v0d2 and DC-STAMP in RAW264.7 cells mediated by RANKL. Therefore, the suppression of pre-osteoclast or osteoclast fusion may be essential for the inhibition of osteoclast differentiation.

## Introduction

Diabetes is becoming a key public health issue due to its increasing prevalence and its effects on several organs, including bone (1). Accumulative evidence has indicated that poor bone quality appears to be the main contributor to the higher risk of fractures observed in either type of diabetes (2). Diabetes is associated with a reduced turnover of bone, with lower levels of bone formation and resorption (3–5), therefore, the poor bone quality in diabetes may be closely associated with reduced bone turnover.

Bone is continuously remodeled by osteoblasts and osteoclasts. Osteoclasts, the only cells capable of resorbing bone, are multinucleated giant cells derived from the monocyte/macrophage lineage. In addition to osteoblasts, the maintenance of normal number and activity of osteoclasts is essential for normal bone turnover. Several previous studies have suggested that high glucose inhibits receptor activator of nuclear factor-κB ligand (RANK)L-induced osteoclastogenesis (6,7). A number of studies investigating type 1 diabetes have observed diminished osteoclast numbers and activity (3,8,9). The suppression of osteoclastic functions has also been found in type 2 diabetes (4,5,10). Therefore, high glucose may inhibit osteoclastogenesis, tip the balance between formation of new bone and resorption of old bone and decrease bone turnover (11). Currently, the specific mechanism involved in the inhibition of osteoclastogenesis remains to be elucidated.

There are three steps in the differentiation of monocyte/macrophage precursors into mature osteoclasts. Initially, monocyte/macrophage precursors become pre-osteoclasts, expressing tartrate-resistant acid phosphatase (TRAP), secondly, the mononuclear pre-osteoclasts fuse together to become nonfunctional multinucleated osteoclasts, which are unable to resorb bone and, finally, the nonfuctional multinucleated osteoclasts are activated into functional osteoclasts (12,13). Osteoclast differentiation depends on RANKL, a TNF family member (14,15). Binding of RANKL to its receptor RANK induces the expression of important transcription factors for osteoclastogenesis, including nuclear factor-κB, c-Fos and nuclear factor of activated T cells c1 (NFATc1) (16–18). Fusion-mediated giant cell formation is essential for osteoclast maturation and is required to resorb bone and the expression of v-ATPase V0 subunit d2 (ATP6v0d2) and dendritic cell-specific transmembrane protein (DC-STAMP) are required for osteoclast fusion (19,20). In the second and third stages of osteoclast differentiation, NFATc1 directly induces the expression of Atp6v0d2 and DC-STAMP, which accelerates cell-cell fusion and the multinucleation process of pre-osteoclasts (13,21).

Diabetic osteoporosis is complex and previous studies have produced conflicting results (3–5,22,23); therefore, further investigation is required at the molecular level of bone in diabetic patients. In the present study, the effects of high glucose on two essential transcription factors and the downstream molecules involved in cell-cell fusion were evaluated.

## Materials and methods

### Cell culture

The RAW264.7 murine monocytic cell line was purchased from KeyGEN Biotech Co., Ltd. (Nanjing, China). The cells were maintained in Dulbecco’s modified Eagle’s medium (DMEM) containing 5.6 mM D-glucose (Hyclone, Logan, UT, USA) supplemented with 100 U/ml penicillin, 100 μg/ml streptomycin and 10% fetal bovine serum (FBS; Hyclone), and incubated at 37°C in 5% CO_2_ humidified air. To investigate the effect of high glucose on osteoclastogenesis, the cells were seeded at a density of 1×10^5^ cells/well in 6-well plates, 2×10^4^ cells/well in 24-well plates and 5×10^3^ cells/well in 96-well plates, and cultured in DMEM with different doses (5.6 and 20.2 mM) of D-glucose or DMEM with mannitol (14.6 mM; Bio Science Technology. Co., Ltd., Shanghai, China) added at 37°C in 5% CO_2_ humidified air for 4 or 5 days. Following attachment of the cells, 100 ng/ml murine recombinant RANKL (PeproTech, Rocky Hill, NJ, USA) was added to the different mediums to generate osteoclasts. The cells cultured with 5.6 mM glucose in the absence of RANKL were defined as the untreated group, those cultured with 5.6 mM glucose in the presence of RANKL were defined as the control group, those cultured with 20.2 mM glucose were defined as the high glucose group and those cultured with mannitol were defined as the osmotic control group.

### Cell viability assay

RAW264.7 cells were seeded at a density of 5×10^7^ cells/well in a 96-well culture plate and cultured in DMEM with various concentrations of D-glucose (5.6 mM, 15.3 mM or 20.2 mM). On day 4, the cell viability was determined using a cell-counting kit-8 (Beyotime Institute of Biotechnology, Haimen, China).

### TRAP staining and TRAP-positive multinucleated cell counting

The cells were stained using an Acid Phosphatase, Leukocyte TRAP kit (387-A; Sigma-Aldrich, St. Louis, MO, USA) to confirm the generation of TRAP-positive cells, which appeared dark red. TRAP-positive multinucleated cells containing three or more nuclei were considered to be osteoclasts. The osteoclasts were then counted and images were captured using a microscope (microscope, JEM1200EX; Camera, Olympus E330; Olympus Corporation, Tokyo, Japan).

### RNA extraction and reverse transcription-quantitative polymerase chain reaction (RT-qPCR)

Total RNA was extracted from the cells with different doses of glucose using the TRIzol method (Invitrogen Life Technologies, Carlsbad, CA, USA) (24), cDNA was synthesized using 1 μg RNA with the PrimeScript RT reagent kit and gDNA Eraser (Takara Bio, Inc., Dalian, China). qPCR was performed using a SYBR Premix Ex Taq reagent (Takara Bio, Inc.) in a Light-Cycler 96 (Roche Diagnostics, Basel, Switzerland) and the primers (Invitrogen Life Technologies, Shanghai, China) used are listed in Table I. The cycles consisted of 95°C for 5 min, followed by 45 cycles of 95°C for 30 sec, 56°C for 30 sec and 72°C for 40 sec. For each sample, the mRNA levels were expressed as a relative value against β-actin mRNA. The relative expression levels of mRNA were calculated as 2^−ΔΔCt^.

### Statistical analysis

Each experiment was performed at least three times. All quantitative data are expressed as the mean ± standard deviation. Statistical analysis was performed with SPSS version 16.0 (SPSS, Inc., Chicago, IL, USA). Statistical differences were analyzed by one way analysis of variance. An LSD test was used for multiple comparisons. P<0.05 was considered to indicate a statistically significant difference.

## Results

### High glucose inhibits RANKL-induced osteoclastogenesis

**Figure 1:**
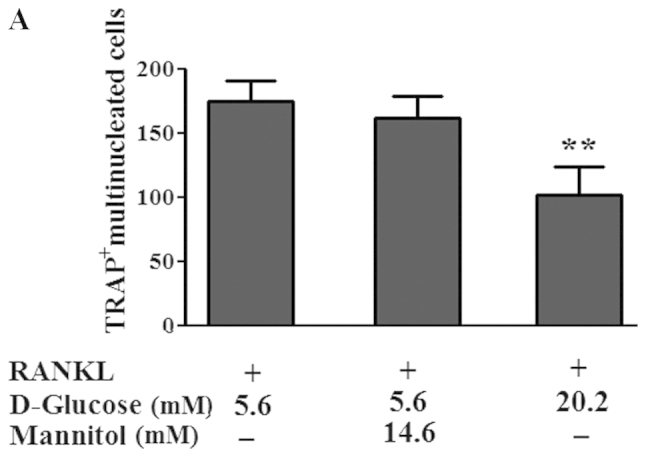

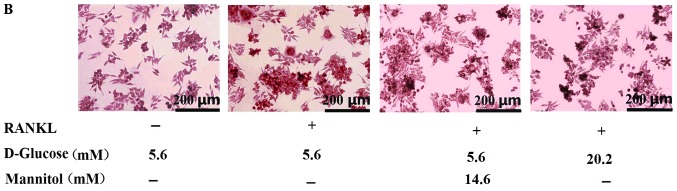
Effect of high glucose on osteoclast formation in RAW264.7 cells. The Raw264.7 cells were seeded at a density of 2×10^4^ cells/well in 24-well plates and cultured with RANKL and different doses (5.6 and 20.2 mM) of D-glucose or mannitol for 4 days. (A) High glucose culture with 20.2 mM D-glucose significantly decreased the number of TRAP-positive multinucleated cells compared with the control culture in 5.6 mM D-glucose (^**^P<0.01; n=4). No significant differences were observed in the osmotic control culture compared with the control culture. Data are expressed as the mean ± standard deviation. (B) Images of the TRAP-positive multinucleated cells were captured under a microscope (magnification, ×200). RANKL, receptor activator of nuclear factor-κB ligand; TRAP, tartrate-resistant acid phosphatase.

**Figure 2:**
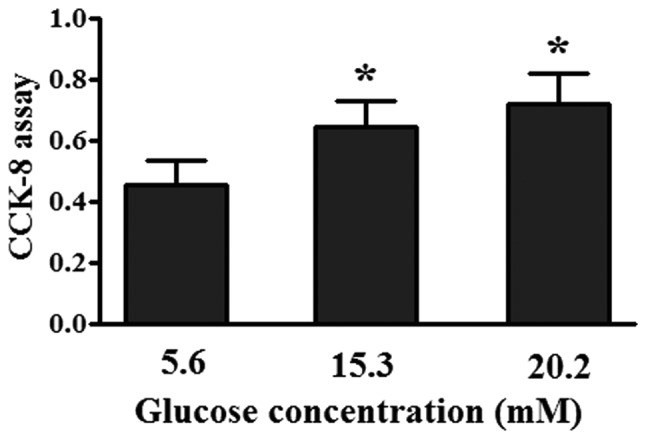
Effect of different concentrations of glucose on cell proliferation. The RAW264.7 cells were seeded in a 96-well culture plate and cultured in Dulbecco’s modified Eagle’s medium with various concentrations of D-glucose, On day 4, the extent of cell proliferation was determined using a CCK-8 assay. Data are expressed as the mean ± standard deviation (n=3). ^*^P<0.05, vs. 5.6 mM glucose-treated cells. CCK-8, cell-counting kit-8 assay.

To examine the effect of high glucose on RANKL-induced osteoclastogenesis, the RAW264.7 cells were incubated with RANKL and different doses of glucose. TRAP staining was then performed to determine the number of osteoclast-like cells. As shown in Fig. 1, RANKL induced the formation of TRAP-positive multinucleated osteoclast-like cells; however, the increase of TRAP-positive multinucleated cells in the control cultures (5.6 mM D-glucose) was inhibited by the addition of 20.2 mM D-glucose. By contrast, no inhibition of RANKL-induced osteoclastogenesis were observed in the cultures incubated with mannitol (osmotic control). TRAP-positive cells appeared dark red in the cytoplasm as shown in the representative images of TRAP-positive multinucleated cells in the different treatment groups in Fig. 1. In order to determine whether the inhibitory effect of high glucose on RANKL-induced osteoclastogenesis was due to its effect on cell growth, a cell proliferation assay was performed. At glucose concentrations of 20.0 mM, high glucose increased cell growth (Fig. 2).

### High glucose suppresses the gene expression of c-fos and NFATc1 in RANKL-induced RAW264.7 cells

**Figure 3:**
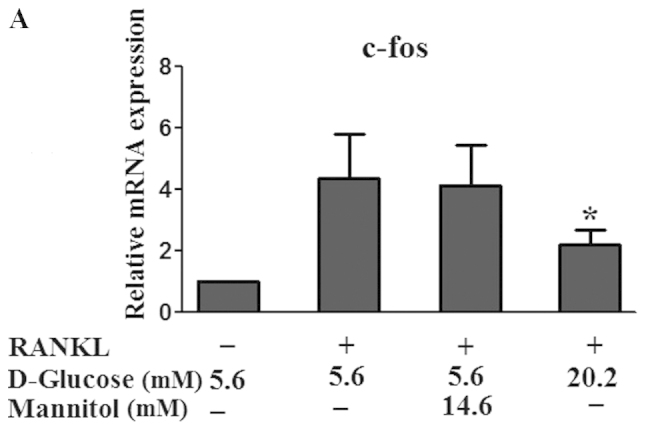

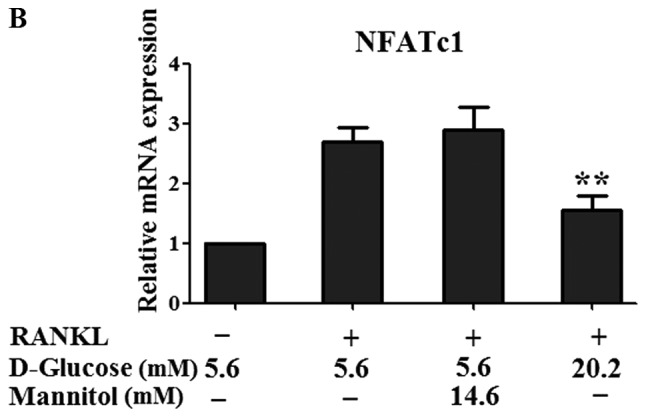
Effect of high glucose on the mRNA expression levels of c-fos and NFATc1. RAW264.7 cells were seeded at a density of 1×10^5^ cells/well in 6-well plates and cultured with RANKL and different doses (5.6 and 20.2 mM) of D-glucose or mannitol for 5 days. The mRNA expression levels of (A) c-fos and (B) NFATc1 were analyzed using reverse transcription-quantitative polymerase chain reaction. Data are expressed as the mean ± standard deviation of the quantification of target expression relative to the untreated group following normalization against β-actin (2^−ΔΔCt^ method). The untreated group were defined as the standard (=1). ^*^P<0.05 and ^**^P<0.01 vs 5.6 mM glucose-treated cells. RANKL, receptor activator of nuclear factor-κB ligand; NFATc1, nuclear factor of activated T cells c1.

RANKL induces the expression of c-Fos and NFATc1 in osteoclastogenesis (25–27) and the transcriptional induction of NFATc1 is a major function of c-Fos in osteoclast differentiation (28). The present study examined the effects of high glucose on the gene expression of c-fos and NFATc1 through RT-qPCR. The addition of high glucose to RAW 264.7 cells cultured in the presence of RANKL downregulated the gene expression of c-fos 2.0-fold (P<0.05) and NFATc1 1.7-fold (P<0.01) as shown in Fig. 3, however, no affect was observed in the gene expression of c-fos or NFATc1 in the cultures incubated with mannitol (P>0.05).

### High glucose inhibits cell-cell fusion in RANKL-induced RAW264.7 cells

Preosteoclast and osteoclast cell-cell fusion is important for the multinucleation of osteoclasts, which is required for their function in bone resorption (13,29). To investigate the role of high glucose in osteoclast fusion, the present study examined the gene expression of ATP6v0d2 and DC-STAMP, which are fusion-mediating molecules. The gene expression levels of ATP6v0d2 and DC-STAMP in the RAW264.7 cells mediated by RANKL increased significantly; however, the mRNA expression of ATP6v0d2 in the RAW264.7 cells cultured with high glucose in the presence of RANKL was significantly inhibited (2.2-fold; P<0.01) and that of DC-STAMP was significant inhibited (1.9-fold; P<0.01).

## Discussion

In diabetes, the bone state is altered, which leads to an increased risk of fracture and a delay in subsequent healing (30). A decrease in the number and the activity of osteoblasts, and reduced bone formation have been found *in vivo* in type 1 and type 2 diabetes (31–33). However, the involvement of bone resorption in diabetes remains controversial. In the present study, high glucose inhibited RANKL-induced osteoclast formation, which was in accordance with the results of certain previous studies (6,7) and high glucose suppressed the expression of key fusion-mediating molecules, possibly by downregulating the expression of c-fos and NFATc1.

The RANKL-RANK axis is essential for osteoclastogenesis (34,35). Binding of RANKL to RANK markedly stimulates the activation of other transcription factors, including activator protein 1 (AP-1) and NFATc1. RANKL activates AP-1 partly by inducing its critical component, c-Fos (16,27,36). Mice lacking c-Fos develop severe osteopetrosis due to the complete inhibition of osteoclast differentiation (37,38). NFATc1 is a key regulator of osteoclastogenesis and is essential for RANKL-induced osteoclast differentiation (27,39). The major function of c-fos in osteoclastogenesis is to trigger a transcriptional regulatory cascade by producing and cooperating with NFATc1, thereby activating certain target genes involved in osteoclast differentiation and function (40). The results of the present study also indicated that RANKL induced the gene expression of c-fos and NFATc1, and high glucose decreased the upregulated expression of c-fos and NFATc1. Therefore, the decrease in the number of multinucleated osteoclast-like cells in high glucose cultures may be associated with the suppression on c-fos and NFATc1 by high glucose.

Osteoclasts are multinucleated, giant cells formed by the fusion of mononuclear pre-osteoclasts (29). Fusion-mediated giant cell formation in osteoclastogenesis is essential for osteoclast maturation and is required for reorganization of the cytoskeleton. Pre-osteoclasts do not resorb bone in *in vitro* cultures and mice with defective pre-osteoclast fusion develop osteopetrosis (19,41), thus, pre-osteoclast fusion is an important cellular event and is required to resorb bone. Among numerous fusion regulators, gene knockout studies have suggested that the expression of Atp6V0d2 and DC-STAMP are required for osteoclast fusion (19,41). Atp6v0d2-deficient mice exhibit impaired osteoclast fusion (20) and DC-STAMP-deficient mice fail to form multinuclear osteoclasts (21). NFATc1 induces osteoclast fusion via upregulation of Atp6v0d2 and DC-STAMP (11), and the expression of DC-STAMP is regulated cooperatively by NFATc1 and c-fos (21). There has been extensive investigation of the regulation of osteoclastogenesis and osteoclast fusion, however, the mechanism by which high glucose regulates the RANKL-mediated signaling pathway and osteoclast fusion requires elucidation. In the present study, the increase of Atp6v0d2 and the expression of DC-STAMP induced by RANKL was inhibited by high glucose, which was likely regulated by c-fos and NFATc1. These results suggested that high glucose inhibited the fusion of pre-osteoclasts.

A previous study attributed the inhibition of osteoclastogenesis in a high glucose environment to a reduction in the production of reactive oxygen species and the activation of caspase-3 and NF-κB (5). The present study suggested that high glucose acted by interfering with RANKL downstream signaling to inhibit osteoclast differentiation and the suppression of RANKL-induced cell-cell fusion may be important.

The decreased formation of multinucleated osteoclasts in high glucose conditions leads to immaturity and a decline in osteoclast function, which results in less efficient removal of damaged bone (42). In addition, osteoclast activity is not restricted to bone resorption. A previous study revealed that osteoclasts may be the source of anabolic signals for osteoblasts and that the initiation of bone formation is likely to be due to osteoclast activity (43), suggesting that the diminished number of osteoclasts in diabetes contributes to a lower number of osteoblasts and, thus, bone formation. Suppression of bone turnover increases the degree of mineralization, which leads to an increase in bone brittleness and subsequent fractures, therefore, osteoclasts are important for sustaining bone quality (44,45).

Several studies have revealed that type 2 diabetic patients have a high fracture rate despite the absence of bone mineral density reduction (46–48). The mechanism involved in the higher fracture risk of diabetes remains to be elucidated. The suppression of osteoclast formation and function by persistent high glucose may contribute to the high fracture rate in diabetic patients and impaired cell-cell fusion may be important, in which the c-fos and NFATc1 signaling pathways may be involved. Techniques to regulate osteoclast differentiation and ameliorate the low bone turnover require further investigation.

In the present study, the expression of two essential transcriptional factors in RANKL-mediated signaling pathway were examined; however, the specific pathway involved in the effects of high glucose remains to be elucidated. Therefore, further investigation is required to identify mechanisms to ameliorate the inhibition of osteoclastogenesis by high glucose.

## Figures and Tables

**Figure 4:**
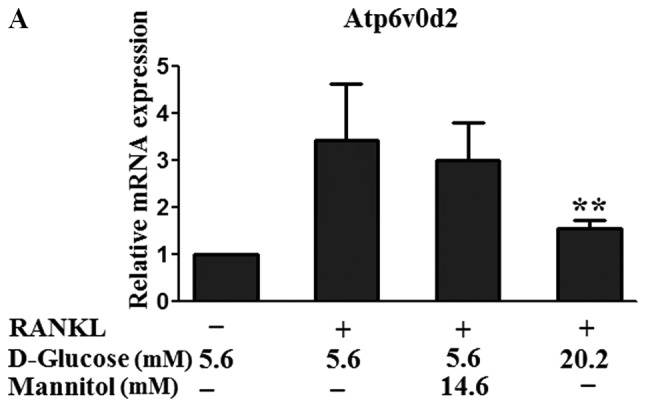

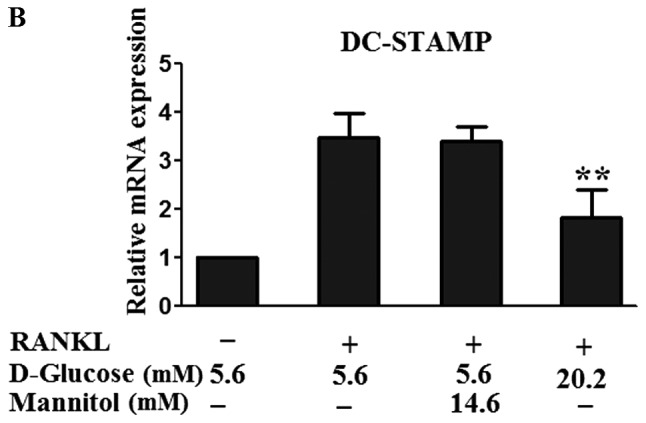
Effect of high glucose on the mRNA expression levels of ATP6v0d2 and DC-STAMP. RAW264.7 cells were seeded at a density of 1×10^5^ cells/well in 6-well plates cultured with RANKL and different doses of D-glucose (5.6 and 20.2 mM) or mannitol for 5 days. The mRNA expression levels of (A) ATP6v0d2 and (B) DC-STAMP were analyzed using reverse transcription-quantitative polymerase chain reaction. Data are expressed as the mean ± standard deviation of the quantification of target expression relative to the untreated group following normalization against the expression of β-actin (2^−ΔΔCt^ method). The untreated group were defined as the standard (=1). ^**^P<0.01, vs. 5.6 mM glucose-treated cells. Atp6v0d2, v-ATPase V0 subunit d2; DC-STAMP, dendritic cell-specific transmembrane protein; RANKL, receptor activator of nuclear factor-κB ligand.

**Table I:** Primers used to amplify cDNA in the present study.

Gene	Primer
Atp6v0d2	Forward: 5′-ATGGGGCCTTGCAAAAGAAA-3′ Reverse: 5′-G CTAACAACCGCAACCCCTC-3′
DC-STAMP	Forward: 5′-GCAAGGAACCCAAGGAGTCG-3′ Reverse: 5′-CAGTTGGCCCAGAAAGAGGG-3′
c-Fos	Forward:5′-CTGGTGCAGCCCACTCTGGTC-3′ Reverse: 5′-CTTTCAGCAGATTGGCAATCTC-3′
NFATc1	Forward: 5′-CAACGCCCTGACCACCGATAG-3′ Reverse: 5′-GGCTGCCTTCCGTCTCATAGT-3′
β-actin	Forward: 5′-GTCATCACTATTGGCAACGAG-3′ Reverse: 5′-CCTGTCAGCAATGCCTGGTACAT-3′

Atp6v0d2, v-ATPase V0 subunit d2; DC-STAMP, dendritic cell-specific transmembrane protein; NFATc1, nuclear factor of activated T cells c1.
